# Evidence that the Amyloid beta Precursor Protein-intracellular domain lowers the stress threshold of neurons and has a "regulated" transcriptional role

**DOI:** 10.1186/1750-1326-3-12

**Published:** 2008-09-02

**Authors:** Luca Giliberto, Dawang Zhou, Richard Weldon, Elena Tamagno, Pasquale De Luca, Massimo Tabaton, Luciano D'Adamio

**Affiliations:** 1 Department of Microbiology and Immunology, Albert Einstein College of Medicine, 1300 Morris Park Ave, Bronx, NY 10461, USA; 2 Department of Experimental Medicine and Oncology, General Pathology Section, University of Turin, Turin, Italy; 3 SZN-BioGeM, Ariano Irpino, Italy; 4 Department of Neurosciences, Ophthalmology, and Genetics, University of Genova, Genova, Italy; 5 Dipartimento di Biochimica e Biotecnologie Mediche, Università di Napoli Federico II, Naples, Italy

## Abstract

**Background:**

Regulated intramembrane proteolysis of the β-amyloid precursor protein by the γ-secretase yields two peptides. One, amyloid-β, is the major component of the amyloid plaques found in Alzheimer's disease patients. The other, APP IntraCellular Domain, has been involved in regulation of apoptosis, calcium flux and gene transcription. To date, a few potential target genes transcriptionally controlled by AID, alone or complexed with Fe65/Tip60, have been described. Although the reports are controversial: these include *KAI1*, *Neprilysin*, *p53, EGFR, LRP *and *APP *itself. Furthermore, *p53 *has been implicated in AID mediated susceptibility to apoptosis. To extend these findings, and assess their *in vivo *relevance, we have analyzed the expression of the putative target genes and of the total brain basal transriptoma in transgenic mice expressing AID in the forebrain. Also, we have studied the susceptibility of primary neurons from such mice to stress and pro-apoptotic agents.

**Results:**

We found that AID-target genes and the mouse brain basal transcriptoma **are **not influenced by transgenic expression of AID alone, in the absence of Fe65 over-expression. Also, experiments conducted on primary neurons from AID transgenic mice, suggest a role for AID in sensitizing these cells to toxic stimuli. Overall, these findings hint that a role for AID, in regulating gene transcription, could be induced by yet undefined, and possibly stressful, stimuli *in vivo*.

**Conclusion:**

Overall, these data suggest that the release of the APP intracellular domain may modulate the sensitivity of neuronal cells to toxic stimuli, and that a transcriptional role of AID could be inscribed in signaling pathways thatare not activated in basal conditions.

## Background

Processing of Amyloid Precursor Protein (APP) by β- and γ-secretase produces Aβ peptides and APP IntraCellular Domain [1,2], the former being the major component of AD amyloid plaques. Recent evidence indicates that AID is a biologically active intracellular peptide. Initial findings indicated that AID could sensitize cells to apoptotic stimuli [3]. Subsequent studies have suggested a role of AID in calcium release from endoplasmic reticulum stores [4]and in gene transcription [5]. The putative transcriptional role of AID has attracted most of the attention because of the functional parallel with Notch signaling, another γ-secretase substrate. In the case of Notch, γ-processing releases NICD that, in the nucleus, binds transcription factors and activates transcription of specific gene targets [6,7]. For APP, similar models have been suggested, where AID travels to the nucleus bound to Fe65 and Tip60 to activate transcription of target genes [5]; furthermore, Fe65 would also boost AID generation [8]. The evidence that AID, Fe65 and Tip60 can all be found on the KAI1 [9] and Neprilysin (NEP) [10] promoters supports this model. AID gene targets that have been described so far include *KAI1 *[9,11], *GSK3 *β [11,12], *NEP *[10], *EGFR *[13], *LRP *[14] and *APP *itself [15], and genes involved in cell cycle control [16] and in tumorigenesis [13]. A genome-wide approach to AID-mediated gene transcription has shown a possible effect of AID in regulating the expression of proteins related to cytoskeletal organization [17] but failed to confirm previous target genes, as have other studies [18,19]. Given this ambiguity in results, we have reexamined the role of AID in transcription and apoptosis *in vivo *studying AID-transgenic (AIDtg) mice. We have found that AID does not univocally regulate the basal expression of *APP, NEP, KAI1 *and *p53 in vivo *in the mouse brain and that the brain transcriptome of AIDtg and littermate mice are identical. Altogether, these findings suggest that a transcriptional role for AID could be inducible. Nonetheless, toxicity tests performed on forebrain primary cortical neurons from AIDtg mice show that AID has the potential to sensitize neurons to toxic stimuli, possibly via a p53-dependent pathway [20,21].

## Materials and methods

### Construction of the transgenic plasmid

The cDNA sequence corresponding to human AID 50, 57 or 59 was subcloned into BamHI-XhoI sites of pHY12 vector, which bears SV40 polyA signal. A NotI-NotI fragment, comprising the transgene, was then cloned into the pNN vector, downstream of the 8-kb CaMKIIα promoter. The whole plasmid was then linearized with SalI, run on agarose gel, purified and injected into oocytes of FVB mice that were than implanted in pseudo pregnant C57BL/6 mice.

### Mice breeding and handling

Mice were maintained on a FVB background and handled according to the Ethical Guidelines for Treatment of Laboratory Animals of Albert Einstein College of Medicine. The procedures were described and approved in animal protocol number 20040707.

### Mice Genotyping

Genomic DNA was extracted and purified from mice tails with DNeasy Tissue Kit (Qiagen), according to the manufacturer's protocol. PCR was conducted using Taq PCR Core Kit (Qiagen) and a Touchdown PCR protocol, starting at 60°C. Primers were constructed on the pNN (fw) and pHY (rev) vectors used for cloning, as follows: fw: 5'-CGAGTGGCCCCTAGTTC-3', rev: 5'-CACTGCATTCTAGTTGTGGTTTG-3'. Internal control primers for β-Actin are as follows: fw: 5'-ACCCACACTGTGCCCATCTA-3'; rev: 5'-CGGAACCGCTACTTGCC-3'. PCR products were run on a 1.5% TBE agarose gel with 0.05% Ethidium Bromide.

### Mouse Brain Dissection

Brains were dissected from sacrificed mice using a 3-diopter magnification lens, in ice-cold, RNase, DNase free 1× PBS (Sigma) made in DEPC double distilled water. One hemisphere, for protein extraction, was shock frozen in liquid nitrogen and stored at-80°C, the other hemisphere was processed for RNA extraction as described. Forebrains only were utilized.

### Primary Neuronal Cultures

Culture plates were coated with 15 μg/mL Poly-L-Ornithine (Low Molecular Weight, Sigma) for 45 minutes at room temperature. Poly-L-Ornithine was the aspirated and wells were soaked with 4 μg/mL mouse Laminin (Invitrogen), for 12–16 hours in a cell culture incubator at 37°C, 95% humidity and 5% CO2. Eight weeks old FVB female mice were bred with age matched male mice for 3 days. Pregnancy was ascertained according to vaginal plug and weight gain of the females. Females were sacrificed by cervical dislocation, after sedation with isoflurane, at 17.5 days of gestation. Foetuses were processed separately, in order to obtain pure transgenic cultures. Genotyping was carried out as described, by isolating tail DNA. Forebrains were dissected in ice cold HBSS (Invitrogen) + 0.5% w/v D-Glucose (Sigma) and 25 mM Hepes (Invitrogen), under a dissection microscope (Zoom 2000, Leica). Dissociation was carried out in ice cold dissection medium plus 0.01%w/v Papain (Worthington), 0.1%w/v Dispase (Roche) and 0.01% w/v DNase (Worthington), first by means of sterile razor blades, then by serial pipetting with flamed sterile glass Pasteur pipettes, and incubation at 37°C twice for 15 minutes. Cells were then spun down at 220 g for 5' at 4°C, resuspended in Neurobasal Medium with 2% B27, 1 mM Na Pyruvate, 100 units/ml penicillin, 100 μg/ml streptomycin, 2 mM Glutamax (all from Invitrogen), filtered through a 40 μm cell strainer (Fisher), counted and plated on coated 6 well plates at a density of about 750.000 cells/well. Culture medium was completely replaced after 16–20 hours, and new medium (30% of starting volume) was added every 3 days until needed. mRNA harvest was performed at 14 and 9 DIV. Also, at 9 DIV, neurons were treated for 3 hours with 500 μM H2O2 in culture medium devoid of Na-Pyruvate, and for 16 hours with either 700 μM Kainic Acid (Sigma), 7 pg/μL FAS Ligand (Upstate), 1 μM Staurosporine (Sigma), 1 μM Aβ 1–42 (Anaspec) or 500 μM Glutamic Acid (Sigma) in their regular culture medium. Also, since replacement of conditioned culture medium with fresh medium determines neuronal suffering in 8 h, and complete death in 30 h, medium was changed 16 hours previous to cell damage and viability tests. All compounds were resuspended, when necessary, according to the manufacturer's instructions, and brought to the desired concentration in sterile double distilled water. Aβ 1–42 was solubilized in Hexafluoroisopropanol (HFIP, Sigma) to 200 μM to prevent aggregation, and stored at -80°C in aliquots. The amount needed was then thawed, HFIP was evaporated under the cell culture hood, and Aβ resuspended in sterile double distilled water to the desired concentration.

### Immunostaining of cultured neurons and transgenic mice brains

Cells, plated on Poly-D-Lysine coated coverslips (24 well plates), were washed in TBS once and fixed with 4% PFA for 30' at room temperature (RT), washed again and permeabilized with 0.2% Triton X-100/TBS for 10' on ice, and cold methanol for 5' on ice. Blocking of aspecific antigenic sites was performed with 10% Goat Serum/0.2% Triton X-100/TBS for 1 hr at RT. Primary antibodies were: anti MAP2 (Sigma, monoclonal clone HM-2, 1/500), anti NeuN (Chemicon, monoclonal clone A60, 1/500), anti GFAP (Abcam 7260, polyclonal 1/500). Secondary antibodies were anti-mouse Alexa Fluor 350 and anti rabbit Alexa Fluor 488, all in 5% Goat Serum/0.1% Triton X-100/TBS for 90' at room temperature. All washes in between and after antibodies incubations were 2 × 10' with TBS pH7.6/0.2% Triton X-100. Coverslips were then mouted on Superfrost Plus(+) glass slides with a glycerol based mount and stored at 4°C, shaded from light. This procedure was optimized in order to obtain maximum reduction of background. Zeiss Axioskop, with fluorescence filters, AxioCam and Axiovision software was used for images acquisition.

### Assessment of neuronal toxicity and viability

Cell suffering was assessed by detecting LDH liberated in the culture medium by damaged neurons treated with toxic/pro-apoptotic stimuli (Roche), according to the manufacturer's instructions. Cell viability was assayed by WST1 incorporation in lively cultured neurons after treatment with toxic/pro-apoptotic stimuli (Roche), according to the manufacturer's instructions.

### AID peptide detection and western blots

Frozen hemispheres were homogenized through sonication (3 × 30" cycles, with 5" pulses) in ice with HU 2–2575 Sonifier (Branson Sonic Power) at #4 power. Buffer is as follows: 2%SDS, 1× Roche Protease Inhibitors Complete Mini-tablets, with EDTA, 5 mM Na3VO4, 50 mM NaF, 1 mM DTT, 1 mM PMSF. Homogenates were spun down at 49000 rpm (100000 g) on a TLA110 rotor (Beckman) at 4°C for 70'. Supernatants, corresponding to 1 mg of total proteins, quantified using BIORAD Smart Spec 3000 and Protein Assay Reagent, were pre-cleared in Protein A Plus (Pierce) for 4 hours at 4°C. Lysates were then incubated with 1 μg of rabbit C-terminal APP antibody (Zymed) over night at 4°C. Finally, Protein A Plus (Pierce) was added again and incubated for 4 hours at 4°C. Beads were washed, resuspended in NuPAGE LDS Sample Buffer/β-MercaptoEthanol, boiled, and 10 μL were loaded on a 4–12% NuPAGE gel. Proteins were then blotted on a 0.2 μm nitrocellulose membrane (Schleicher & Schuell), blocked in 5% milk/PBS and probed with either rabbit anti APP C-terminal antibody (Zymed, 1/500 dilution) or with the rabbit C8 antibody (provided by Dennis Selkoe, 1/500 dilution). Western Blots on **homogenates **from AIDtg and littermate mice were carried out as described previously [22]. Secondary antibody was a goat anti rabbit-HRP (Southern Biotech, 1/3000 dilution). C8 was diluted in Superblock/PBS (Pierce), while secondary antibody was dilute in 5% milk/PBS. Blots were developed with SuperSignal West Pico Chemiluminescent Substrate (Pierce) and SuperSignal West Dura Extended Duration Substrate (Pierce).

### RT and Quantitative PCR

Each experiment was done in triplicate. Several primer pairs were tested, prior the experiments, to check for proper amplification and to rule out primer dimerization. Selected primers are as follows:

-hsAID: fw: 5'-GCATCGATTCTAGAATTCG-3'; rev: 5'-CCACCACACCATGATGAAT-3'

-hsAPP: fv: 5'-TCGGAAGTGAAGATGGATGC-3'; rev: 5'-CCTTTGTTCGAACCCACATC-3'

-mmKAI1: fv: 5'-CCTCTTCCTCTTCAACTTGCT-3'; rev: 5'-CGGAAATGAAGCTGTTCTTG-3'

-mmNeprilysin: fw: 5'-GGACATGAAATCACACATGG-3'; rev: 5'-AAATTATTTGCCGACTGCTG-3'

-mmβ-actin: fv: 5'-AAATCGTGCGTGACATCAAA-3; 5'-TCTCCAGGGAGGAAGAGGAT-3'.

**Mouse brain mRNA **was extracted with Trizol reagent (Invitrogen), processed and purified with RNeasy Protect Kit (Qiagen) according to the manufacturers' protocols. Two μg of RNA were retro transcribed to cDNA using SuperScript III First-Strand Synthesis System for RT-PCR kit (Invitrogen). Quantitative PCR was carried out using Power Sybr Green PCR Master Mix on a ABI PRISM 7900 HT Sequence Detection System (Applied Biosystems) according to the manufacturer's protocols. Data analysis was conducted according to M. W. Pfaffl [23] and Applied Biosystems references and protocols.

### Sample preparation and hybridization for micro array analysis

Each experimental point was performed in triplicate. Mouse Brains were homogenized in TRIZOL reagent (Invitrogen) and extracted following the manufacturer's protocol. A further purification step with the PROTECT kit (Qiagen) was added. cRNA was generated by using the Affymetrix One-Cycle Target Labeling and Control Reagent kit (Affymetrix Inc., Santa Clara, California, USA), following the manufacturer's protocol. The biotinylated cRNA was hybridized to the MOE 430 2.0 Affymetrix DNA chips, containing over 39000 genes and open reading frames from *M. musculus *Genome databases GenBank, dbEST and RefSeq. Chips were washed and scanned on the Affymetrix Complete GeneChip^® ^Instrument System, generating digitized image data files.

### Micro array data analysis

DAT files were analyzed by MAS 5.0 for detection calls (Affymetrix Inc.) and RMA for expression values. The expression values obtained were analyzed by using GeneSpring GX (AgilentTechnologies). Results were filtered for flag (presence call), then for fold change > 1.5, obtaining a total of 5019 probe sets differentially expressed in the samples versus the controls. Statistical analysis was initially performed using the Two-Way ANOVA using Age and Transgene Expression as parameters to test. As Age was the only parameter to give significant results, we next applied a Welch T-Test on Age using as p-value cutoff 0.001, multiple testing correction Bonferroni, obtaining a set of 380 genes statistically significant. Transgene Expression didn't give any significant result even using a p-value cut-off 0.05.

### Statistical analysis

All quantified data represent an average of at least triplicate samples. Error bars represent standard errors of the mean. Statistical significance was determined by Student's t test and a p < 0.05 was considered significant.

## Results

### Generation of AID transgenic animals

**Figure 1:**
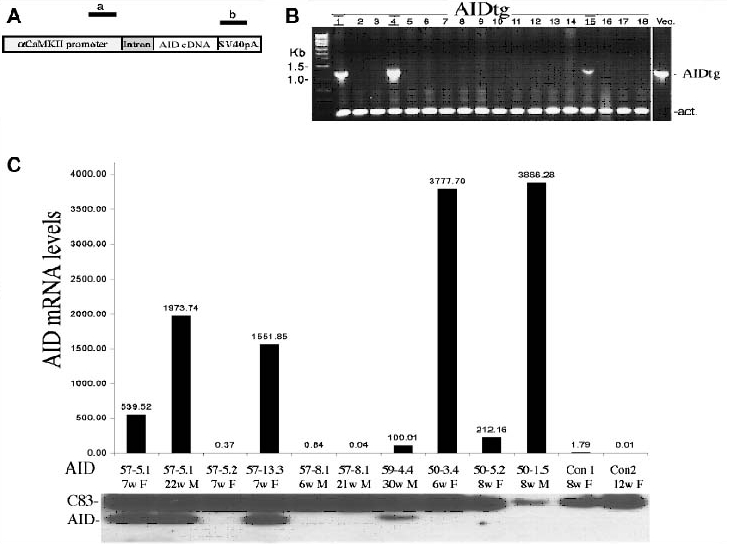
**Characterization of AID transgenic mice**. **A **Schematic representation of the transgenic AID constructs (fragments are not depicted in scale). The location of the PCR primers a and b used for genotyping is shown. **B **PCR of 63 pups (18 are shown here) revealed that 9 of them (three are shown here, number 1, 4 and 15) had integrated the AID transgene. In the same PCR tube, β-actin was amplified to control for genomic DNA content. Vec. represents the control PCR performed using the transgenic vector as a template. **C **Real Time PCR showing the expression levels of the *AID *transgene in different lines. **D **Immunoprecipitation and western blot was conducted from a brain hemisphere of AIDtg and littermate (Con) mice. Mice lines that expressed the AID protein at the mRNA level, and had a detectable band at the western blot analysis were selected for further studies.

To directly examine the effects of AID in vivo, and in the brain, we generated transgenic mice expressing AID under the control of the CaMKIIα promoter, targeting its expression to the forebrain regions (which comprise the thalamus, hypothalmus and the upper telencephalon) of the postnatal mouse [24]. These areas are most relevant to Alzheimer's pathology. Endogenous AID is very short lived and therefore virtually undetectable [25]. We generated transgenic lines expressing either the 59- or 57-residue AID peptide, which would be produced by γ-cleavage together with either Aβ40 or Aβ42, respectively. In addition, transgenic lines expressing a "ε-cleavage" AID of 50-residue [26,27] were also made. AID cDNAs were cloned downstream of the 8-Kb CaMKIIα promoter and into a plasmid containing a mini-intron and the SV40 polyadenylation sequence [24] (Figure 1A). The linearized plasmids were injected into oocytes of FVB mice that were than implanted in pseudo pregnant C57BL/6 mice. Tail-DNA PCR, showed that 9 out of 63 pups obtained had integrated the *AID *transgenes (samples are shown in Figure 1B). More specifically, we obtained two AID59 (AID59-4.4 and -1.1), four AID57 (AID57-13.3, -5.1, -5.2 and -8.1) and three AID50 (AID50-3.4, -1.5 and 5.2) founder mice. Germline transmission was observed for all founders. The expression levels of the *AID *transgene mRNA and protein were determined by real-time quantitative PCR and Immunoprecipitation followed by Western blot analysis, respectively. Total RNA and protein lysates were isolated from the forebrain of adult AIDtg animals. Different levels of *AID *mRNA and AID peptide were found in the different transgenic mice (compare Figure 1C and 1D). Of note, AID50-1.5 and AID50-3.4 lines expressed the highest levels of *AID *mRNA but no detectable AID50 protein. This data suggests that AID50 is the more unstable AID peptide form.

All mice show, up to 24 months of age, a regular growth pattern and mating ability, and we cannot detect any gross deficit or behavioral abnormality among the different AIDtg lines compared to the wild type littermates.

### APP, KAI1, NEP and p53 gene expression is not altered in AID transgenic adult animals

**Figure 2:**
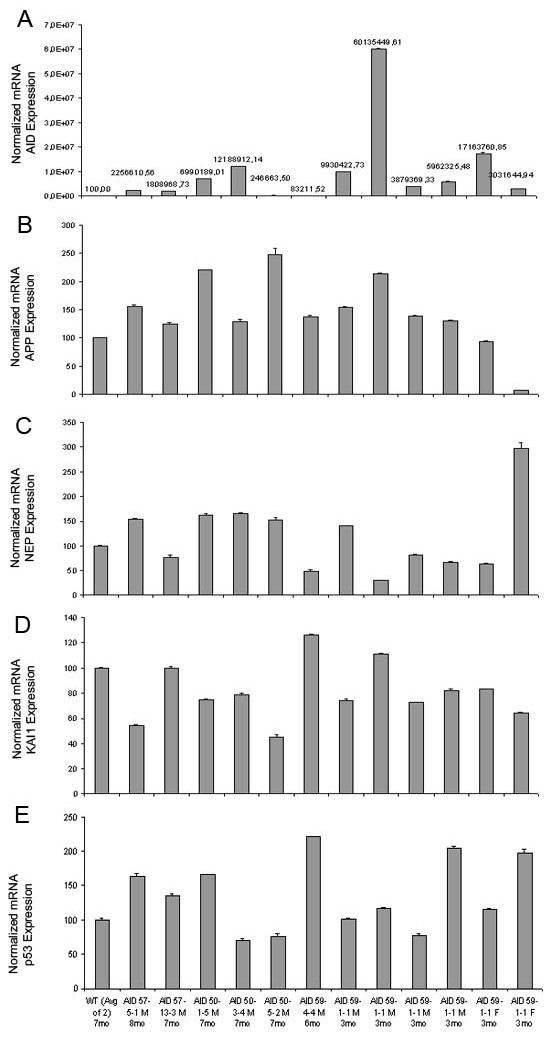
**In vivo expression of candidate AID targets is not affected by transgenic AID expression, in adult mice**. Real Time Quantitative PCR shows the relative expression of *AIDtg *protein, **A**, *APP*, **B**, *NEP*, **C**, *KAI1*, ***D***, *and p53*, ***E ***in the forebrain and hippocampus of AIDtg and their littermate control mice. Values are relative to 100% value given arbitrarily to a littermate mouse. Experiments were conducted in triplicate loading, and the error bars represent standard deviations.

To determine whether AID affects *APP, KAI1, NEP *and *p53 *mRNA expression *in vivo *in the brain, RNA from the forebrain of adult (3–8 months) AID57-5.1, AID57-13.3, AID59-4.4, AID59-1.1, AID50-1.5, AID50-3.4, AID50-5.2 and control littermates were analyzed by real-time quantitative PCR. The data presented in Figure 2 show that there is no obvious correlation between *AID *mRNA and *APP, KAI1, NEP *and p53 levels, considering also age, sex and AID transgene levels of expression. Overall, these data suggest that AID is not involved in the basal expression of putative AID transcriptional targets in the adult mouse forebrain.

### AID does not regulate basal gene expression in the mouse brain

**Figure 3:**
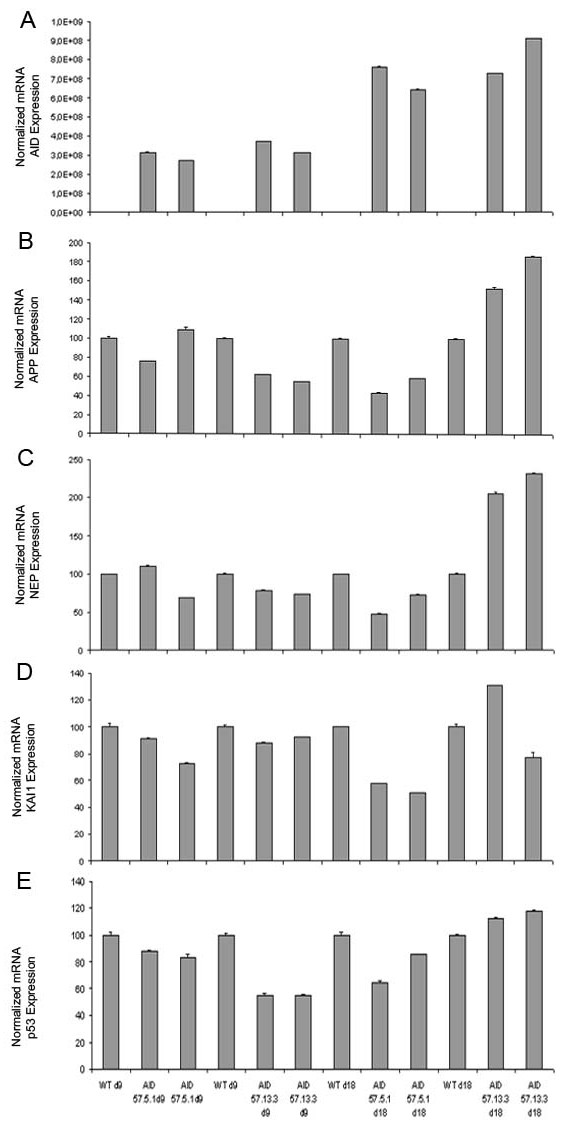
**AID is already expressed at postnatal day nine, but does not influence the expression of the candidate genes**. Real Time Quantitative PCR shows the relative expression of *AIDtg *protein **A**, *APP*, **B**, *NEP*, **C**, *KAI1*, ***D***, *and p53*, ***E ***in the forebrain and hippocampus of AIDtg and their littermate control mice as early as 9 and 18 days post-natal. For *APP, NEP*, *KAI1 *and *p53 *expression, values are relative to 100% value given arbitrarily their day 9 and day 18 littermates. For *AIDtg *protein, 100% value was assigned to the first day 9 littermate mouse. Experiments were conducted in triplicate loading, and the error bars represent standard deviations.

A role for AID in transcription cannot be nonetheless excluded from the above evidence. AID might indeed regulate transcription of yet unidentified genes. To address this point we took advantage of our AID tg mice. It is foreseeable that an AID target should be dis-regulated in the forebrain of AID tg mice. RNA from the forebrain of AID59-4.4, AID57-5.1, AID57-13.3 and control littermates was prepared from either 9 days or 18 days old mice. AID50tg mice were not included in this analysis because we could not detect expression of the AID50 peptide. Nine day old animals were selected as further negative controls because the transgenic cassette should be expressed only two weeks after birth. However, we detected expression of *AID *mRNA before day 18, in the 9 days old AIDtg (Figure 3). Before using these samples for micro array analysis, the RNAs were tested for *APP, KAI1, NEP *and *p53 *expression. Once more, we saw no clear-cut correlation between expression of AID and that of its putative gene targets (Figure 3, AID 57-5.1 and 13.3 shown). Since we did not test *EGFR *and *LRP *mRNA levels, those genes (even their basal transcription) may still be regulated by AID alone, without over-expression of Fe65. Regardless, we analyzed the forebrain transcriptome of these mice using an Affymetrix DNA chips, containing over 39000 genes and open reading frames from *M. musculus *Genome databases GenBank, dbEST and RefSeq. Statistical analysis performed using age and transgene expression as parameters to test, showed that age difference was the only parameter to give significant results, yielding a set of 380 genes that were differentially expressed between 9 and 18 day old mice (data not shown), indicating changes in the transcriptome during post-natal development. Transgene expression didn't give any significant result even using a p-value cut-off 0.05 indicating that the forebrain transcriptome was identical in all age-matched mice analyzed. The above data argue against a role for AID in basal transcriptional regulation.

### Transgenic AID expression is detectable in fetal neurons in culture, does not influence the expression of target genes, but increases neuronal sensitivity to toxic and apoptotic stimuli

**Figure 4:**
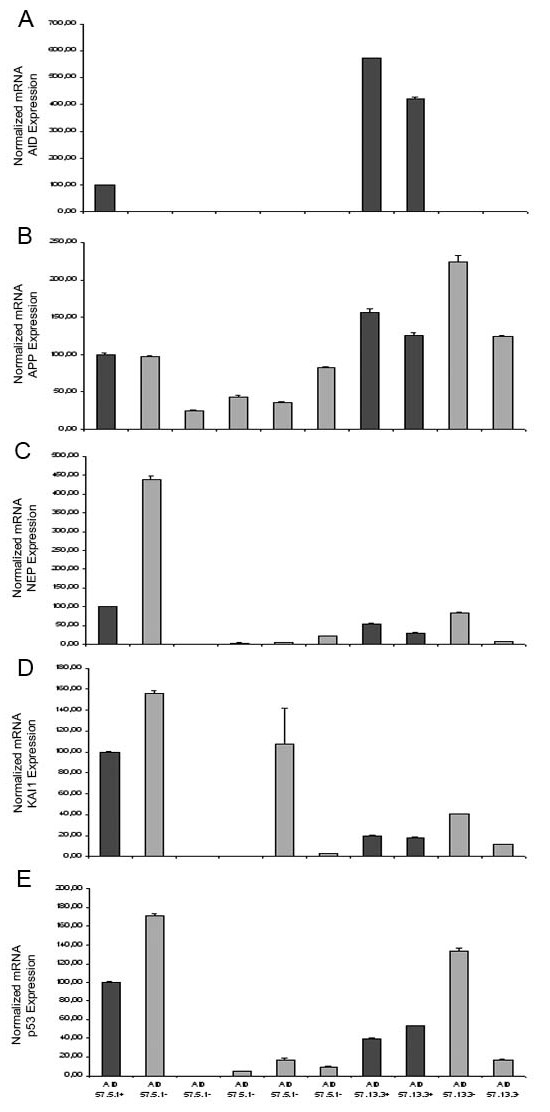
**AIDtg expression in cultured fetal primary neurons does not change the relative expression of *APP, NEP, KAI1 and p53***. **A ***AIDtg *expression was confirmed by tail genotyping of fetuses (not shown) and by QPCR data on cultured neurons (dark bars). Expression of *AID*, **A**, *APP*, **B**, *NE*P, **C**, *KAI1*, **D**, and *p53*, **E**, is relative to the 100% value given arbitrarily to the first *AIDtg *mouse. Experiments were conducted in triplicate loading, and the error bars represent standard deviations. Cultures were harvested at DIV 14. Similar results were achieved from younger cultures (DIV 9, not shown) and in the *AIDTg 59 *line (not shown).

The finding that TgAID, under the control of our forebrain promoter, is expressed even at postnatal day 9, has led us to think that we could exploit the potentiality of neuronal cultures to assess the role of AID. Neurons from embryonic day 17.5 fetuses, cultured for 9 days, showed detectable TgAID expression (Figure 4A). As for postnatal and adult mice though, the presence of AID 57 (data not shown for AID 59) did not seem to influence the relative expression of our target genes (Figure 4B–E).

**Figure 5:**
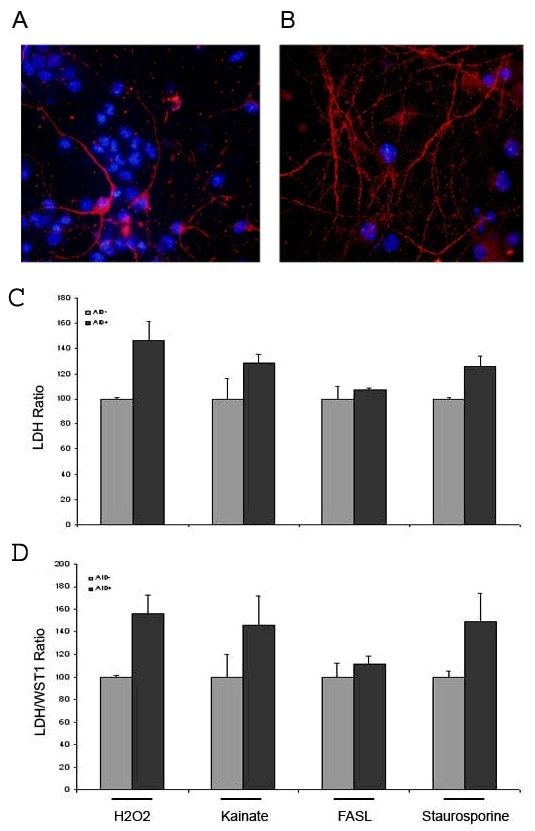
***AIDtg *expression in cultured fetal primary neurons increases their sensitivity to toxic and apoptotic stimuli**. *AIDTg *line 59.4.4, at DIV 9. To verify the purity of the neuronal cultures, cells were stained with anti-NeuN (Blue) plus anti-GFAP (red), **A**, and NeuN (Blue) plus anti-MAP2 (red), **B**. **C **Toxicity of indicated stimulus was assessed by measuring LDH release. **D **Released LDH was weighed against WST-1 uptake in the LDH/WST-1 ratio, which confirms the trend of LDH release. **C **and **D **Values from toxic stimuli were weighed against values from untreated cells to express the increase of the indicators of cell damage. Average values refer to at least 3 AIDtg and 3 littermate mice; measurements were done on 6 separate wells of 96 well culture plate for each foetus' neurons. Similar results were obtained for *AIDTg *57 line (not shown).

Since AID has been implicated in pathways leading to cell death and apoptosis [3,12,28] we aimed to determine its role under cellular stress conditions. We prepared neuronal cultures from AID59 (and AID57, not shown) mice and littermates. Purity of these cultures was assessed by staining for Microtubule Associated protein 2 (MAP2), Neuronal Nuclei (NeuN) and Glial fibrillary Acidic Protein (GFAP) (Figure 5A and 5B). MAP2 is the major microtubule associated protein of brain tissue, is known to promote microtubule assembly and to form side-arms on microtubules. It also interacts with neurofilaments, actin, and other elements of the cytoskeleton. It electively stains dendrites. NeuN (or Neuronal Nuclei) reacts with most neuronal cell types throughout the nervous system of mice including cerebellum, cerebral cortex, hippocampus, thalamus. Developmentally, immunoreactivity is first observed shortly after neurons have become postmitotic. The immunohistochemical staining is primarily localized in the nucleus of the neurons. GFAP is a member of the class III intermediate filament protein family. It is heavily, and specifically, expressed in astrocytes and certain other astroglia in the central nervous system. Antibodies to GFAP are therefore very useful as markers of astrocytic cells.

As shown in figure 5C, AID59 (analogous results for AID57, not shown) positive neurons have a lower threshold to cell damage induced by toxic or pro-apoptotic stimuli, as indicated by LDH release in culture medium. In particular, H2O2, Kainate and Staurosporine show the biggest differences in LDH release between AID positive and negative cells; the biggest difference in cell viability is seen in Kainate and Staurosporine treatments. When 1 μM Aβ 1–42, 500 μM Glutamate and "starvation" were used as noxious stimuli, no difference was detected between transgenic and non-transgenic neurons (data not shown). As expected, there was no difference in these indicators, between AID positive and negative neurons, in non-treated cells (not shown). In figure 5D, LDH release was weighed with WST-1 incorporation in the same cells. It is possible that transcription of genes involved in apoptosis, e.g., p53 etc, may be regulated by AID under the stress or pathological conditions.

## Discussion and Conclusion

The findings that AID might regulate apoptosis and Ca++ flux were met with some skepticism. On the contrary, hints to a transcriptional role of AID generated great enthusiasm given the parallel with Notch signaling. Several reports have pointed to few possible AID transcriptional targets. The evidence that AID is found on the *KAI1 *promoter, where it is perhaps complexed to Fe65 and the hystone acetyltransferase Tip60 [9], have supported a direct role for AID in transcription. It can also be hypothesized that AID regulates transcription indirectly and that APP functions as a "hanger" that restrains Fe65 outside the nucleus: APP processing would activate transcription, as it liberates Fe65 and allows it to translocate to the nucleus. More recent data have suggested that the APP/Fe65 interaction promotes a "conformational maturation" of Fe65 that is converted into a transcriptionally active state [29]. However, the reported AID-dependent changes in gene expression has been questioned [18]. Therefore, we have reexamined the role of AID in *in vivo *transcription. Overexpression of AID in the mouse brain did not affect the levels of these three putative AID gene targets. Although these findings question the role of AID in basal transcription of these candidate genes, it is still possible that authentic AID targets genes have not been yet characterized. To address this we have analyzed the effect of AID expression on the mouse brain transcriptoma. The data show that the gene expression pattern of AIDtg and littermate mice is identical, failing to identify any other potential AID gene targets. Thus, AID might have a transcriptional function either in a small subset of forebrain neuronal cells, in cell types different than those analyzed here or under specific signaling or stressful conditions. Genome-wide analysis, conducted on neuronal cells expressing inducible AID, has shown that several genes involved in cytoskeletal dynamics can be regulated by AID. The finding has been confirmed, by SYBR Green real-time PCR, in brains of AD patients for 2-Actin, IGFBP3, and TAGLN [17]. These target genes do not seem to be regulated by AID in our model. This might be due to two reasons. Induction of the AID transgene was allowed for 72 hours in culture before any effect could be detected. Our mice overexpressed AID for several days, as also evident by AID mRNA detection in cultured neurons. It is foreseeable that any effect of AID overexpression, during a longer period of time and in a more complex setting, as is the living mouse brain, would probably result in a different expression arrangement, especially of genes devoted to maintaining the integrity of the cell. Also, sporadic AD brains are a much more complex and entropic system than ours, allowing for complex interactions between different pathogenic entities. Thus, we cannot exclude that a dis-regulation of these target genes may happen later in the life of our mice or under different stress conditions. The role of the intracellular fragment of APP, could possibly be understood by studying its effect under specific stress situations, e.g. under apoptotic or oxidative stimuli, where it could play either a protective or a detrimental role for the cell, depending on other factors such as cell types and interaction with other proteins. This would also explain the predisposition of some brain regions to Alzheimer's pathology. AID has been proposed as a possible mediator of cell death, via a reduction of the cellular threshold to apoptosis [3]. But recent findings have also pointed to a possible protective effect of the APP c-terminal/Fe65 interaction, involving DNA damage response [28]. Our data show that over-expression of AID in cultured neuronal cells predisposes them to a higher degree of suffering, i.e. to a lower resistance to toxic and apoptotic stimuli. In our hands, only selective stimuli could reveal this peculiarity, possibly because of different threshold to cell damage for each experimental compound. Recent works show a role for AID in *p53 *associated cell death [10]. In our model, under toxic stimuli, AID may lower the threshold to cell death through a p53-dependent mechanism by augmenting p53 expression. However, further experiments are required to test this hypothesis.

We believe that the key to understand the role of APP processing in gene regulation lays in the complex interaction of APP domains with other intra- or extra-cellular factors, possibly having a role only in certain stressful situation or at a given "age". Further work will explore the nature of this complex network.

## Competing interests

The authors declare that they have no competing interests.

## Authors' contributions

LG participated in the design of the study, handled the mice colony and genotyping, designed the experiments, performed most of the experiments and cultures, participated in the final analysis and draft preparation. DZ participated in the mice colony handling and genotyping, and characterization of tg mice. RW participated in the mice colony handling and genotyping. ET participated in the design of the study. PDL performed all the micro-array experiments and analysis. MT participated in the design of the study. LD conceived and designed the study, designed the tg mice, participated in the design of the experiments, participated in the handling of the mice colonies and genotyping, and in the analysis of the data, prepared the draft.
